# The importance of pollen chemistry in evolutionary host shifts of bees

**DOI:** 10.1038/srep43058

**Published:** 2017-02-20

**Authors:** Maryse Vanderplanck, Nicolas J. Vereecken, Laurent Grumiau, Fabiana Esposito, Georges Lognay, Ruddy Wattiez, Denis Michez

**Affiliations:** 1 Laboratory of Zoology, Research Institute for Biosciences, University of Mons - UMONS, Place du Parc 20, B-7000 Mons, Belgium; 2 Landscape Ecology & Plant Production Systems Unit, Interfaculty School of Bioengineering (EIB), Université Libre de Bruxelles, Boulevard du Triomphe CP 160/12, B-1050 Brussels, Belgium; 3 Département de Biologie des Organismes, Université Libre de Bruxelles, av. Paul Héger, CP 160/12, B-1050 Brussels, Belgium; 4 Earth and Life Institute, Biodiversity Research Centre, Université Catholique de Louvain, Croix du Sud 4 L7.07.04, B-1348 Louvain-la-Neuve, Belgium; 5 Unit of Analytical Chemistry, University of Liège (Gembloux Agro-Bio Tech), Passage des Déportés 2, B-5030 Gembloux, Belgium; 6 Department of Proteomic and Microbiology, Research Institute for Biosciences, University of Mons - UMONS, Place du Parc 20, B-7000 Mons, Belgium

## Abstract

Although bee-plant associations are generally maintained through speciation processes, host shifts have occurred during evolution. Understanding shifts between both phylogenetically and morphologically unrelated plants (i.e., host-saltation) is especially important since they could have been key processes in the origin and radiation of bees. Probably far from being a random process, such host-saltation might be driven by hidden constraints associated with plant traits. We selected two clades of oligolectic bees (i.e., *Colletes succinctus* group and *Melitta leporina* group) foraging on co-flowering but unrelated host-plants to test this hypothesis. We analyzed floral scent, floral color and chemical composition of pollen from host and non-host plants of these two clades. We did not find evidence for host-plant evolution in the *Melitta leporina* group driven by one of the assayed floral traits. On the contrary, hosts of the *C. succinctus* group display similar primary nutritive content of pollen (i.e., amino acids and sterols) but not similar floral scent or color, suggesting that shared pollen chemistry probably mediates saltation in this clade. Our study revealed that constraints shaping floral associations are diverse and clearly depend on species life-history traits, but evidence suggests that pollen chemistry may act as a major floral filter and guide evolutionary host-shifts.

Like many phytophagous insects^1^, bees display a high diversity in host-plant use and taxonomic range. Bee species can be pollen specialists foraging on a few related plant species belonging to the same family (i.e., oligolectic species), whereas other species display a wider taxonomic range including at least two plant families (i.e., polylectic species) (for review and definitions, see ref. 2). Studies combining bee phylogeny and host-plant use have highlighted that shifts in host-plants in groups of pollen specialist species have occurred several times independently in the course of evolution, even if phylogenetic conservatism in host-plant associations following speciation processes was also documented^2^,^3^,^4^,^5^,^6^. Different patterns have been described for bees that shifted host-plants: While species are predominantly found to shift towards host plants belonging to the same plant tribe (e.g., *Andrena* bees, see ref. 5), other phylogenetic trait mapping studies have provided evidence for shifts between morphologically rather than phylogenetically related host-plants^6^,^7^,^8^,^9^. Interestingly, some bee clades have undergone shifts between both phylogenetically and morphologically unrelated host-plants (e.g., *Capicola, Hesperapis, Melitta* and *Colletes*)^6^,^10^. Although the mechanisms underlying these broad shifts (i.e., host-saltation) remain unidentified, they are likely far from being a random process. Just as specialized species cannot exploit several hosts because of pollen chemistry and floral traits (e.g., color, scents, morphology)^11^,^12^, similar physiological or neurological constraints probably mediate the evolutionary patterns of host-plant use^9^,^13^. Among floral traits used by bees for selecting their hosts, floral scents appear to play a major role in long- and short-distance attraction^14^,^15^,^16^. These olfactory cues are also associated with visual stimuli, including floral color detected in three receptors by bees (UV, blue, green)^17^,^18^,^19^. The combination of the two stimuli seems to drive specialized interactions between bees and the flowers they visit^20^ (but see ref. 21 on a bee-flower interaction based on sexual deception).

Besides these sensory stimuli, pollen traits such as its structure (i.e., exine thickness) or its chemistry (i.e., the pollenkitt and the pollen chemical contents) could also drive bee female host-plant choices^11^,^22^,^23^. These traits vary highly among plant species, and some may lead to physiological limitations related to pollen digestion, lack of essential nutrients or toxic compounds^11^,^23^,^24^,^25^,^26^,^27^,^28^. The effects of natural concentrations of toxins on bees are often quite mitigated, detrimental but rarely lethal and sometimes beneficial (i.e., reduction of parasitism and predation)^29^,^30^,^31^,^32^. By contrast, natural concentrations of nutrients (i.e., sterols, amino acids and polypeptides) impact many essential biological processes such as growth, development, immuno-competence and reproduction^33^,^34^,^35^,^36^,^37^. Moreover, phytosterols were described as stimuli of foraging behavior in the Western honeybee, *Apis mellifera*^38^,^39^. All these chemicals may potentially be drivers of bee evolutionary shifts and are obviously detected by bees^40^,^41^,^42^,^43^,^44^,^45^.

Physiological and neurological constraint hypotheses in host-plant use are supported by recent experimental studies on specialist wild bees^2^,^12^,^23^, but their involvement in evolutionary host switching is still unknown. By analyzing the evolution of host choices through pollen and floral traits in two selected clades of specialist bees, the *Melitta leporina* group (Apoidea, Melittidae, three species) and the *Colletes succinctus* group (Apoidea, Colletidae, three species), we provide the first study on evolutionary drivers of host shifts. Specifically, we investigated whether host-plants foraged by closely related bee species associated with morphologically and phylogenetically unrelated plant families share similarities in their floral chemistry and/or color. Understanding these patterns of host-saltation is especially important since they could have been key processes in the origin and early radiation of bees.

## Results

### Floral scent

**Figure 1:**
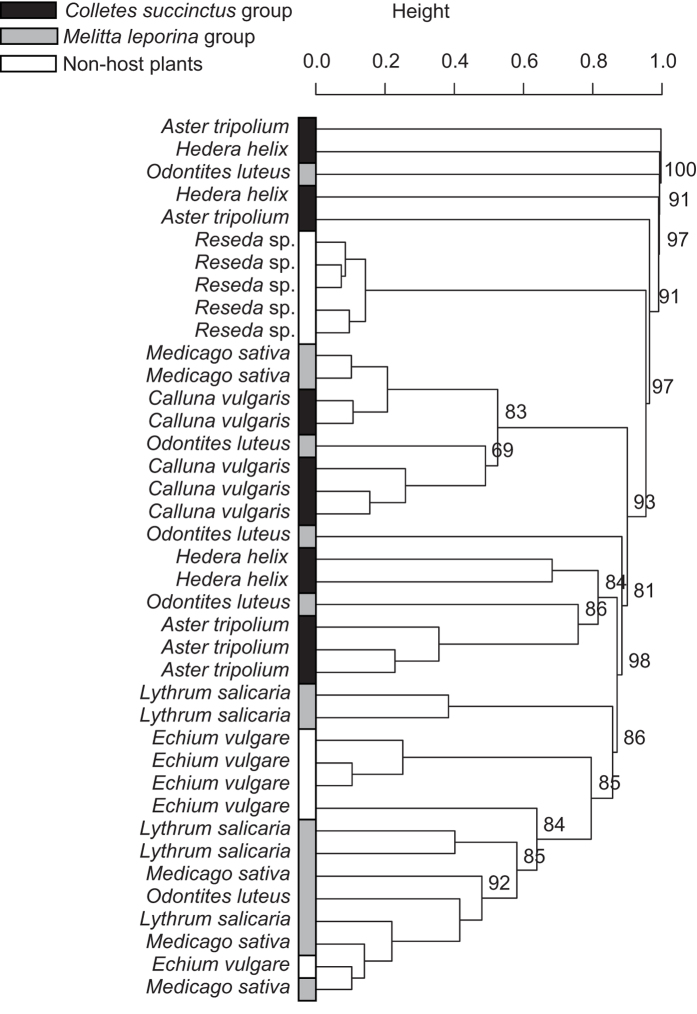
Floral scents. UPGMA cluster based on Bray-Curtis dissimilarity index of floral volatile compounds (relative abundances, in %). Color refers to the non-host plants (white) or to host plants of either the *Colletes succinctus* group (black) or the *Melitta leporina* group (grey). The values near nodes are multiscale bootstrap resampling, and only values of main groups are shown.

We detected a total of 80 volatile compounds from the odor samples (Supplementary Table S1 online). Air and vegetative control samples did not contain quantifiable amounts of any compounds assessed. Each plant species emitted distinct floral scents (F_7,31_ = 4.90, *p* = 0.001; multiple pairwise comparisons *p* < 0.05), except *Medicago sativa*, which was not significantly different from *Lythrum salicaria* (F_1,8_ = 2.27, *p* = 0.06), *Odontites luteus* (F_1,8_ = 1.25, *p* = 0.299) and *Calluna vulgaris* (F_1,8_ = 4.17, *p* = 0.064); as well as *Aster tripolium*, which was not significantly different from *Hedera Helix* (F_1,7_ = 1.62, *p* = 0.096). Despite these similarities, all the host-plants of both *C. succinctus* and *M. leporina* groups were scattered across the whole cladogram (Fig. 1). Moreover, cluster analysis highlighted the intraspecific variability with some plant species clearly spread across the cladogram (e.g., *Aster tripolium, Lythrum salicaria, Medicago sativa* and *Odontites luteus*) (Fig. 1). No significant association between any volatile compound of floral scents and any plant species was detected.

### Floral reflectance

**Figure 2:**
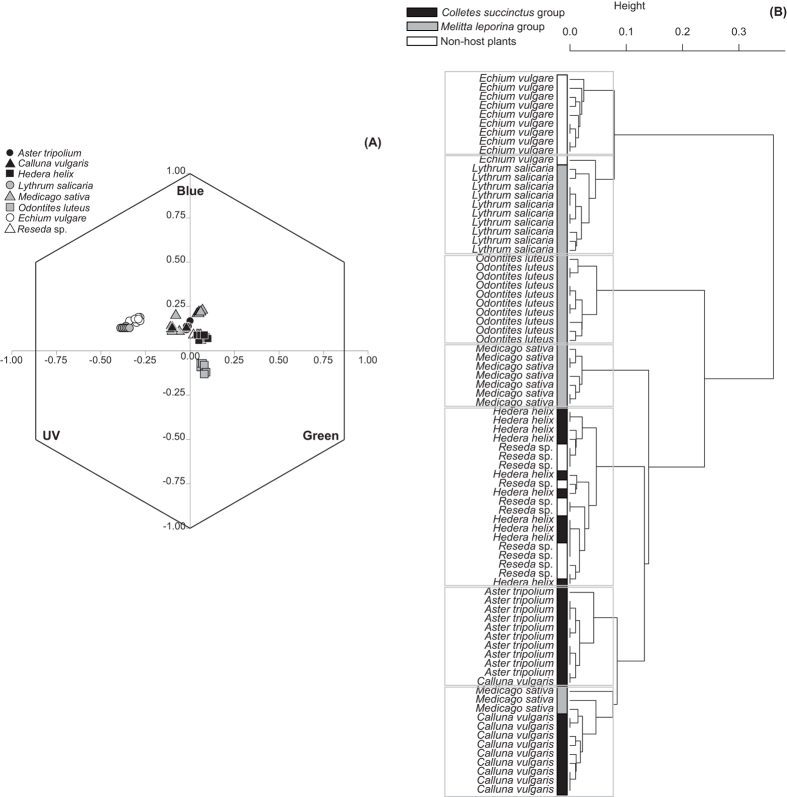
Floral reflectance. UPGMA cluster based on Euclidean distance matrix (**B**) based on XY coordinates of the individual spectral loci calculated for the bee color hexagon (**A**). Color refers to the non-host plants (white) or to host plants of either the *Colletes succinctus* group (black) or the *Melitta leporina* group (grey). The values near nodes are multiscale bootstrap resampling, and only values of main groups are shown.

Spectral reflectance analyses indicate that the plants exploited by sister species of bees are not more similar to one another than expected by chance (F_2,77_ = 3.03, *p* = 0.052). For example, floral spectra of *Hedera helix* (host plant of *Colletes hederae*) and *Reseda* sp. (non-host plant) are more similar to one another (i.e., they form a mixed cluster in Fig. 2; F_1,18_ = 0.43, *p* = 0.555) than other host plants within the *Colletes succinctus* species group (*Hedera helix* versus *Aster tripolium,* F_1,18_ = 103.77, *p* = 0.001; *H. helix* versus *Calluna vulgaris*, F_1,18_ = 191.69, *p* = 0.001). Likewise, *Lythrum salicaria* was found to cluster apart from the other host plants of the *Melitta leporina* group (Fig. 2) (i.e., *Medicago sativa* and *Odontites luteus*), suggesting again that flower spectral similarity as perceived by the bees is unlikely to have driven shifts in host plant use per se.

### Pollen chemical content

**Figure 3:**
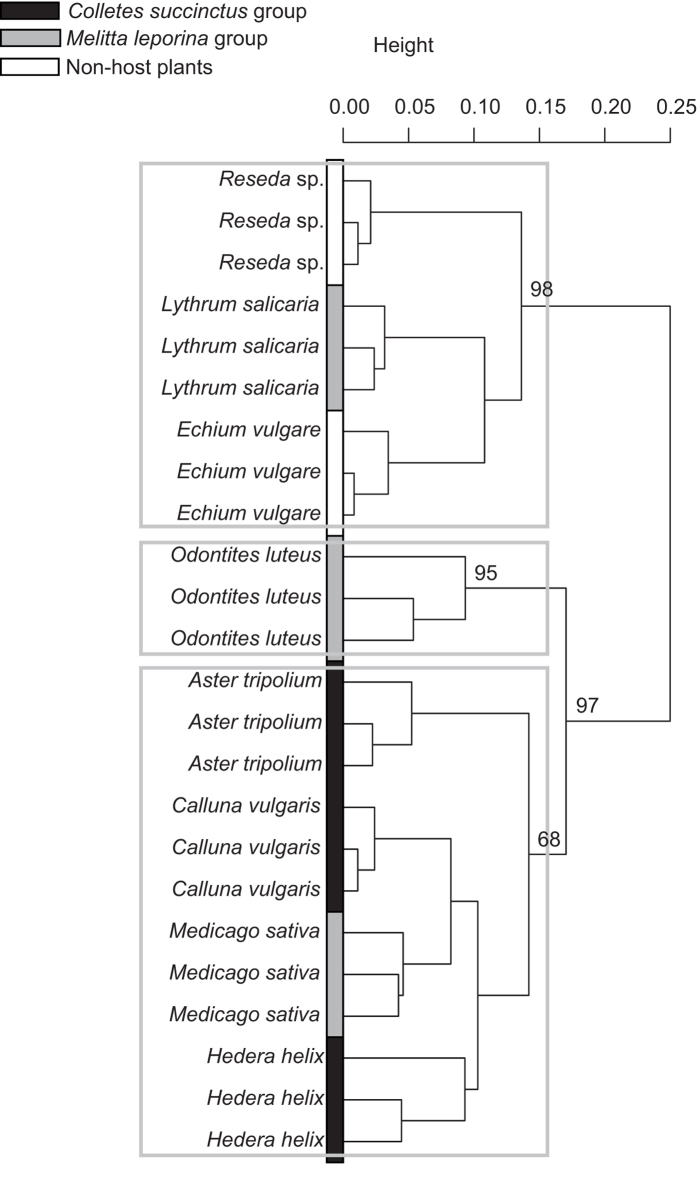
Pollen chemistry. UPGMA cluster based on Bray-Curtis dissimilarity index of global chemical composition of pollen (i.e., sterol, polypeptide and amino acids). Color refers to the non-host plants (white) or to host plants of either the *Colletes succinctus* group (black) or the *Melitta leporina* group (grey). The values near nodes are multiscale bootstrap resampling, and only values of main groups are shown.

The analyses of pollen chemistry show that samples grouped together according to plant species (Fig. 3). Furthermore, the host-plants of *Colletes succinctus* group are clustered, whereas the host-plants of *Melitta leporina* group are scattered across the whole cladogram (Fig. 3). Such gathering of host-pollen of the *C. succinctus* group is partly due to their similar polypeptide content surrounding 135–140 mg/g (post-hoc test, *p* > 0.05) (Table 1) compared to the variable polypeptide amount of host-pollen of *M. leporina* group (post-hoc test, *p* < 0.05) (Table 1). Phytosterolic composition seems also to support the host-plant cluster of *C. succinctus* group because the occurrence of δ7-avenasterol in pollen is indicative of this group (Indicator Compound Analysis, *p* = 0.011, indicator value = 0.629) despite variation in sterolic composition among the three host-pollens (F_2,6_ = 271.89, *p* = 0.004) (Supplementary Table S2 online). By contrast to polypeptide and sterol contents, no clear discrimination of host and non host-plants was found based on amino acid content of pollen (Table 1). Amino acid profile appeared quite conserved among the plant species because no significant association between any amino acid and any plant species or bee group was detected (Supplementary Table S3 online).

## Discussion

Our results show that the composition of amino acids (including the full spectrum of essential amino acids) appears quite similar regardless of the plant species. This result is consistent with previous studies suggesting that amino acid profiles are highly conserved among plants^34^,^46^. There is growing evidence that this trait could be an adaptive response of plants to ensure pollinator attraction^43^.

By contrast, polypeptide and sterolic contents of pollen are quite variable among species. Whereas *Lythrum salicaria* pollen displays only 32 mg/g of polypeptides, *Odontites luteus* pollen shows greater quality, with 226 mg/g of polypeptides. Such variability has been already highlighted among different plant species^47^ and seems to be correlated with protein content of the pollenkitt^48^. Both protein content of pollen^49^ and pollenkitt (i.e., pollen coat proteins)^48^ obviously account for floral preferences of pollinators. In particular, rich-protein pollenkitt renders pollen attractive to animals and provides a digestible reward for pollinators. Moreover, pollenkitt is involved in pollen stickiness, which enables adhesion to insect bodies and pollen packaging by bees (i.e., pollen transport)^22^.

As in many plants, the pollen of *Echium vulgare, L. salicaria, O. luteus* and *Reseda lutea* show high levels of 24-methylenecholesterol, β-sitosterol and δ-5 avenasterol^35^,^50^,^51^,^52^. This similarity of pollen composition among phylogenetically distant plants could allow bees to evolve a more generalist foraging behavior and then promote generalization in pollination systems. By contrast, host-plants of *C. succinctus* group display particular pollen sterolic profile with less common sterols such as δ7-sterols. These uncommon sterolic compounds could filter through the available spectrum of floral visitors and thereby promote tight association with obligate specialists. Such specialization in pollination systems presents advantages for both bees and plants since it reduces pollinator competition and improves plant pollination by restricting the range of visitors to a specialist guild^53^.

Little is known about the functional significance of sterol diversity. One hypothesis is that sterol profiles may reflect adaptations to local abiotic conditions, but this explanation was not always sufficient^35^. Another hypothesis is that phytosterol profiles may function as a unique defense against insect herbivores such as grasshoppers^35^. Sterolic composition of pollen may therefore play a dual role as a nutritional compound for effective pollinators and a toxic repellent for herbivores, robbers or non-effective visitors.

Growing evidence suggests that pollination syndromes are not limited to morphological traits, but that convergent suites of floral chemical traits could also act as filters in host plant selection and therefore pollination systems^46^,^54^,^55^. Pollen nutritional content is obviously a key element for understanding host-plant choices and their evolution among bees.

Pollen from phylogenetically and morphologically unrelated plants associated with *C. succinctus* group displays strikingly similar nutritional profiles with 135–140 mg/g of polypeptides and presence of δ7-sterols. Choices of alternative host-plants appear therefore as a non-random process as these three closely related colletid species seem to share similar physiological (i.e., biological processes) and/or neurological (i.e., pollen recognition) constraints in their polypeptide requirement and appear able to metabolize quite rare δ7-phytosterols, which could lead to tight bee-plant association. Such use of δ7-phytosterols has been already highlighted in planthoppers that produce 24-methylenecholesterol from ergosta-5,7,24(29)-trienol by using a δ7-reductase from intracellular yeast like symbiotes^56^. Evidence indicates that bee species of the *C. succinctus* group might be physiologically rather than neurologically limited to exploit alternative flowers because their host-plants are highly divergent in color and scent but produce pollen with similar chemical composition.

In an evolutionary context, such similar pattern in pollen nutrients suggests that species from *C. succinctus* group inherited from a common ancestor the abilities to successfully utilize their host-pollens (i.e., preadaptation by evolutionary retention). Müller and Kuhlmann^2^ have postulated that the ancestor of the *C. succinctus* group might possibly have been an Asteraceae oligolege that passed on the physiological capability to utilize Asteraceae pollen to the contemporary species. Species from *C. succinctus* group might therefore be expected to be able to feed on their original host. This assumption is supported by Müller and Kulhmann^2^, who showed that *C. hederae* and *C. succinctus* occasionally harvest pollen on hosts already utilized by their respective sister species (i.e., Asteraceae). The same pattern was found in butterflies of the tribe Nymphalini, which are able to feed on *Urtica*, probably their ancestral host regardless of their actual host-plant^57^. There is also evidence that adaptation to new hosts need not preclude use of ancestral host types in *Osmia californica*^26^.

Pollen nutritive content is highly variable among the Fabaceae, Lythraceae and Orobranchaceae species investigated, with regard to polypeptide concentration (i.e., from 32 to 226 mg/g) and sterolic composition (i.e., any indicator compound associated with hosts of *Melitta leporina* group). Such selection of chemically divergent host-pollens suggests that the nutritional profile of pollen does probably not influence the host-plant choices in this bee group. Compared to *C. succinctus* group, the species of *Melitta leporina* group obviously display a higher physiological plasticity that could be promoted by either existence of pre-adaptations^58^, symbiosis with particular microorganisms^59^,^60^,^61^,^62^ or Dufour’s gland secretions^63^. In particular, Dufour’s gland of bees is known to be an extremely rich source of diverse natural products, which are mostly used for lining the brood cell and communication^63^. The assumption that hypertrophied Dufour’s gland may be involved in melittid tolerance to nutritional variations is supported by the larval nutritional function of Dufour’s gland secretions described in *Anthophora, Emphoropsis* and *Megachile* bees^64^,^65^. Although *M. leporina* group does not appear physiologically constrained in terms of floral preference, another mechanism, possibly not atavistic, might have driven the incorporation of novel hosts.

The co-flowering and co-occurrence of an alternative host within the geographical range of the ancestral host has probably facilitated colonization and then incorporation of novel hosts by the three closely related melittid species^6^. The assumption that floral associations in the *M. leporina* group have been driven by partial overlap of spatial and temporal distributions is strongly supported by the widespread distributions of contemporary *M. leporina* hosts. Furthermore, the oligolectic *Melitta* genus has been already regarded as an ecological opportunist in previous studies^66^,^67^. It is currently assumed that such a mechanism would allow the insect to expand its geographical range into the areas where the novel host grows, but where the ancestral host does not^58^.

Though floral choices in specialist bees appear to be a dynamic process, we provide evidence that it is not a highly flexible trait as chemical filters (e.g. pollen nutritive content) can guide evolutionary host-shifts like in *C. succinctus* group. However our study revealed that constraints shaping floral associations are highly diverse and clearly depend on species life-history traits as ancestral events themselves are defining trait that allows colonization of novel host and subsequent host-switch.

## Material and Methods

### Bee species and their host-plant associates

**Figure 4:**
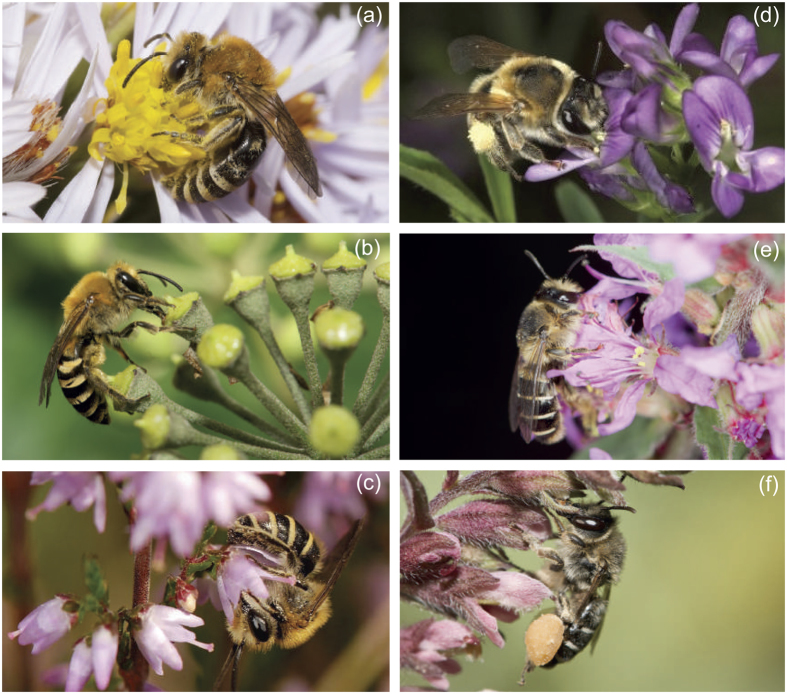
Oligolectic bee species and their host-plants. On the left, the *Colletes succinctus* group with (**a**) *C. halophilus* on *Aster tripolium* (Asteraceae), (**b**) *C. hederae* on *Hedera helix* (Araliaceae) and (**c**) *C. succinctus* on *Calluna vulgaris* (Ericaceae). On the right, the *Melitta leporina* group with (**d**) *M. leporina* on *Medicago sativa* (Fabaceae), (**e**) *M. nigricans* on *Lythrum salicaria* (Lythraceae) and (**f**) *M. tricincta* on *Odontites verna* (Orobranchaceae). Photographs by Nicolas J. Vereecken.

We focused our study on two clades of bees: the *Melitta leporina* group (Apoidea, Melittidae) and the *Colletes succinctus* group (Apoidea, Colletidae) and their host-plants (Fig. 4).

Melittidae is a basal family in the bee clade^27^. It mainly includes oligoleges and is therefore key to shedding light in the early steps of floral specialization and host choices among bees^67^. We selected three sister species within *Melitta leporina* group, namely, *Melitta leporina* (Panzer), *Melitta nigricans* Alfken and *Melitta tricincta* Kirby^68^,^69^. Females restrict their pollen collection to a limited number of related plant species, but they display different host ranges. *M. leporina* is a broad oligolege on the very common plant family Fabaceae^6^. *M. nigricans* and *M. tricincta* collect pollen exclusively on the genera *Lythrum* (Lythraceae) and *Odontites* (Scrophulariaceae), respectively^6^ (Fig. 4). These two plant genera display broad continental distribution but are only locally abundant. We selected three host-plants for this group: *Lythrum salicaria (M. nigricans* host), *Medicago sativa (M. leporina* host) and *Odontites luteus (M. tricincta* host).

Colletidae is a derived family with many generalist species and some oligoleges^2^,^27^. Among these species, we selected three close relatives belonging to the *Colletes succinctus* group, namely, *Colletes halophilus* Verhoeff, *Colletes hederae* Schmidt and Westrich and *Colletes succinctus* (L.)^10^. All these species have been long classified as strictly oligolectic, but their specialization degrees were revised by Müller and Kuhlmann^2^. Whereas *C. halophilus* is always considered a strict Asteraceae specialist preferring flowers of the Asteroideae^2^,^10^ (Fig. 4), *C. hederae* and *C. succinctus* appear more flexible in pollen-host choices^2^. Although *C. hederae* and *C. succinctus* prefer to collect pollen on *Hedera* (Araliaceae)^70^,^71^ and on Ericaceae, respectively^72^ (Fig. 4), both species seem able to harvest pollen on the main host of their respective sister species. This peculiar foraging behavior makes the *C. succinctus* group a promising candidate for elucidating mechanisms of the evolution of host-plant choices in specialist bees. We selected three host-plants for this group: *Aster tripolium (C. halophilus* host), *Calluna vulgaris (C. succinctus* host) and *Hedera helix (C. hederae* host).

As groups for comparison, we selected two plant species, namely, *Echium vulgare* (Boraginaceae) and *Reseda lutea* (Resedaceae), which are melitophilous, co-flowering and co-occurring species with host plants previously described but that are not exploited by *Melitta leporina* or *Colletes succinctus* group^2^,^6^.

All these eight plants (hosts and non-hosts) display contrasting floral morphologies and are phylogenetically distant^73^. Although their habitats are quite different, they are potential hosts for the six selected bee species since they are all present in Western Europe and can be in bloom in the same time (i.e., summer- and autumn-flowering species)^2^,^66^,^74^,^75^.

### Floral scents

Floral scent emitted from the host plants of the *Melitta leporina* group (*Lythrum salicaria* (Lythraceae), *Odontites luteus* (Orobranchaceae) and *Medicago sativa* (Fabaceae)), from the host-plants of *Colletes succinctus* group (*Aster tripolium* (Asteraceae), *Calluna vulgaris* (Ericaceae) and *Hedera helix* (Araliaceae)) and from co-flowering non-host plants (*Echium vulgare* (Boraginaceae) and *Reseda* sp. (Resedaceae)) were collected from five plants per species grown in a garden at the University of Mons (Belgium) and in neighboring semi-natural sites. All the plants were visited by local wild bee populations and can therefore be considered attractive. We used dynamic headspace adsorption during the peak flowering time of these taxa from July to August 2014. The freshly opened inflorescences were enclosed in polyacetate oven bags (Toppits^®^); we used on average three inflorescences for each sample and a total of 5 replicates for each plant species. The air and the floral volatiles were trapped in Teflon-PTFE cartridges (60 mm × 3 mm id) containing 85 mg of the adsorbent Tenax-GR using a battery-operated membrane pump at a flow rate of 100 ml.min-1. This adsorbent was exposed to the flower fragrance for 2 hours during daytime. Ambient controls were collected from empty bags and vegetative controls from leaves following the procedure as described above. We then eluted the trapped scent compounds with 200 μl of cyclohexane and stored at −20 °C until analysis by Gas Chromatography/Mass Spectrometry (GC-MS) (Supplementary Appendix S1 online).

Differences in floral scents were assessed using perMANOVA (Bray-Curtis dissimilarity index, 999 permutations, “adonis” command) and multiple pairwise comparisons with Bonferroni’s adjustment after testing for multivariate homogeneity (“betadisper” command) (R-package vegan, see ref. 76). Differences were visually assessed on UPGMA clusters using Bray-Curtis dissimilarity index and multiscale bootstrap resampling to calculate p-values for uncertainty in hierarchical cluster (R- package pvclust, see ref. 77). Indicator compound analyses were also performed to identify floral scent compounds that were indicative of host-plants in a particular bee group (“indval” command) (R-package labdsv, see ref. 78). All these analyses were conducted in R version 2.15.1^79^ using data expressed as relative abundances.

### Floral reflectance

A portable spectrophotometer (AVASPEC-2048- USB2-UA; Avantes, Eerbeek, The Netherlands) equipped with a Xenon light source (AVALIGHT-XE; Avantes) was used to measure the relative reflectance (in %, 300–700 nm in 5-nm steps) of ten flowers/inflorescences of each plant species (Appendix S1) following the method described in Vereecken *et al*.^80^. The spectrophotometer was calibrated with a white standard (WS-2; Avantes) prior to the measurements. We used the spectral sensitivity functions of the honeybee (*Apis mellifera*) because they are largely consistent within bees^81^,^82^. We then converted the raw data (relative reflectance measurements, in %) into individual loci in the bee color hexagon^18^ by using the honeybee receptor-sensitivity curves and a green leaf background. We assessed and quantified the color and achromatic contrasts between floral spectra by calculating (a) pairwise Euclidean distances between loci and (b) the mean Euclidean distance between the species centroids in the bee color hexagon. The Euclidean distance between any two loci indicates the perceived color difference or contrast between the stimuli, and threshold values of hexagon units for color discrimination usually range between 0.062^83^,^84^,^85^ and 0.100^86^ for bees.

To test the hypothesis that closely related bee species are specialized on host plants that are visually more similar to one another than can be expected by chance alone, we performed perMANOVA analyses followed by post-hoc comparisons (Bonferroni’s adjustment) using 999 permutations and a pairwise Euclidean distance matrix based on the XY coordinates of the individual spectral loci calculated above for the bee color hexagon. Differences were visually assessed on a UPGMA cluster using Euclidean distances. This approach allowed us to test the extent to which the floral spectra as perceived by the bees were clustering together according to the two clades of specialist bees investigated.

### Pollen chemical content

We collected an equivalent quantity of pollen (100 mg) for each target plant (Supplementary Table S4 online). We used a tuning fork to remove pollen from flowers and cleaned the samples under a binocular microscope before lyophilization. The different pollen samples were then stored at −20 °C. The polypeptide content was assessed from five milligrams of each pollen species in triplicate following the method described in Vanderplanck *et al*.^87^. Quantifications were performed three times for each extraction using standard curve of BSA and BCA Protein Assay Kit (Pierce, Thermo Scientific). Amino acids were analyzed from three samples of 3–5 mg pollen for each plant species as described in Vanderplanck *et al*.^87^. Total amino acids were measured separately with an ion exchange chromatograph (Biochrom 20 plus amino acid analyzer) using norleucine as internal standard. Only tryptophan was omitted because its isolation requires a separate alkaline hydrolysis from additional amounts of sample, and it is hardly ever a limiting essential amino acid^88^. Sterol content was analyzed from three samples of twenty milligrams of each pollen species according to the method described in Vanderplanck *et al*.^89^. The total sterol contents were determined considering all peaks of sterols (upper the LOQ) eluted between cholesterol and betulin. Individual sterols were quantified on the basis of peak areas from analyses. Identifications were achieved by comparing the relative retention times (ß-sitosterol –TMS = 1.00) with those of oil reference (sunflower oil with well-known composition). These identifications were checked by GC/MS (Gas Chromatograph/Mass Spectrometer) analyses^89^.

We conducted the same statistical analyses as those used for floral scents to assess significant difference in pollen compositions (perMANOVA and multiple pairwise comparisons) and to detect chemicals that are statistically associated with certain bee groups (indicator compound analyses). Dissimilarities in pollen chemicals among plant species were visualized on a UPGMA cluster using Bray-Curtis dissimilarity index and multiscale bootstrap resampling. The statistical analyses were conducted on data expressed as concentrations in mg/g.

## Additional Information

**How to cite this article**: Vanderplanck, M. *et al*. The importance of pollen chemistry in evolutionary host shifts of bees. *Sci. Rep.* **7**, 43058; doi: 10.1038/srep43058 (2017).

**Publisher's note:** Springer Nature remains neutral with regard to jurisdictional claims in published maps and institutional affiliations.

## Supplementary Material

Supplementary Information

## Figures and Tables

**Table 1:** Polypeptide, amino acid (i.e. total and essential amino acids) and sterol content of host and non-host pollens expressed as means (sd).

Bee group	Pollen source	Polypeptide content (mg/g)	Amino acid content (mg/g)		Sterol content (mg/g)
			Total	Essential	
*Colletes succinctus*group	*Aster tripolium* (n = 3)	139 (17.7) d	220.8 (7.5) a	111.2 (3.9) a	3.3 (0.2) a
	*Calluna vulgaris* (n = 3)	133.7 (9.3) d	290 (2.5) ab	145.7 (1.1) ab	9.8 (0.6) d
	*Hedera helix* (n = 3)	135.6 (13.7) d	325.4 (41.2) bcd	144.4 (19.9) abc	4.2 (0.3) bc
*Melitta leporina*group	*Lythrum salicaria* (n = 3)	32.2 (1.3) b	315.2 (10.5) abcd	150.8 (4.6) abc	16.9 (2.4) e
	*Medicago sativa* (n = 3)	177.1 (17.9) e	301.5 (13.5) abc	149.3 (6.5) abc	4.8 (0.3) c
	*Odontites luteus* (n = 3)	225.9 (14.3) f	363.3 (57.6) cd	167.6 (26.2) bc	4.8 (0.4) c
Outgroup	*Echium vulgare* (n = 3)	14.9 (0.8) a	361.2 (11.8) d	175.9 (6.9) c	3.6 (0.3) ab
	*Reseda lutea* (n = 3)	84.5 (1.8) c	342.7 (5.8) bcd	156.4 (4.1) bc	8.5 (0.9) d
Statistics		***F***** = 407.46 *****P***** < 0.001**	***F***** = 7.12 *****P***** < 0.001**	***F***** = 4.41 *****P***** = 0.007**	***F***** = 129.55 *****P***** < 0.001**

Values with the same letter are not significantly different.
