# Supplementary material for: The importance of pollen chemistry in evolutionary host shifts of bees
Source: Sci Rep. 2017 Feb 20;7:43058. doi: 10.1038/srep43058 (PMC5316986; doi:10.1038/srep43058)
Supplement: Supplementary Information [file srep43058-s1.pdf]

# – Supplementary Information –

The importance of pollen chemistry in evolutionary host shifts of bees

Maryse Vanderplanck[1,*], Nicolas J. Vereecken[2], Laurent Grumiau[3], Fabiana Esposito[4], Georges Lognay[5], Ruddy Wattiez[6] and Denis Michez[1]

[1] Laboratory of Zoology, Research Institute for Biosciences, University of Mons - UMONS, Place du Parc 20, B-7000 Mons, Belgium.

[2] Landscape Ecology & Plant Production Systems Unit, Interfaculty School of Bioengineering (EIB), Université Libre de Bruxelles, Boulevard du Triomphe CP 160/12, B-1050 Brussels, Belgium.

[3] Département de Biologie des Organismes, Université Libre de Bruxelles, av. Paul Héger, CP 160/12, B-1050 Brussels, Belgium.

[4] Earth and Life Institute, Biodiversity Research Centre, Université Catholique de Louvain, Croix du Sud 4 L7.07.04, B-1348 Louvain-la-Neuve, Belgium.

[5] Unit of Analytical Chemistry, University of Liège (Gembloux Agro-Bio Tech), Passage des Déportés 2, B-5030 Gembloux, Belgium.

[6] Department of Proteomic and Microbiology, Research Institute for Biosciences, University of Mons - UMONS, Place du Parc 20, B-7000 Mons, Belgium.

[*] Corresponding author; +32 67 35 38 03; maryse.vanderplanck@umons.ac.be

Supplementary Appendix S1. Details on the Gas Chromatography/Mass Spectrometry (GC-MS) procedure used to determine floral scents

The extracts were analysed by Gas Chromatography/Mass Spectrometry (GC-MS) on a Thermo Trace GC Ultra coupled to a Thermo Polaris-Q ion trap mass and equipped with a Restek RXI-5 MS column (30 m length x 0,25 mm diameter x 0,25 μm film thickness). Aliquots (1 μl) of the extracts were injected in splitless mode with an initial temperature of the column maintained 4 min at 35 °C and then programmed to 200 °C at 5 °C min^{-1} and maintained for 1 min. After this isotherm at 200 °C, the temperature was increased to 270 °C (10 °C min^{-1}) then isothermal at 270 °C for 10 min. Helium was used as carrier gas. We used the Xcalibur ™ Software to calculate the relative amounts of odor compounds in the floral scent extracts.

Supplementary Table S1. Floral scent compositions from the host-plants of the *Colletes succinctus* group, the *Melitta leporina* group and the plant species of the outgroup. Mean ± SE, relative amounts (in %).

Compounds	***Colletes succinctus* group**			***Melitta leporina* group**			**Outgroup**	
	Aster tripolium (n = 5)	*Calluna vulgaris* (n = 5)	*Hedera helix* (n = 4)	*Lythrum salicaria* (n = 5)	*Medicago sativa* (n = 5)	*Odontites luteus* (n = 5)	*Echium vulgare* (n = 5)	*Reseda lutea* (n = 5)
Fatty acid derivatives (%)	39.91 ± 19.38	81.40 ± 7.57	0	11.87 ± 6.68	49.08 ± 20.41	52.73 ± 17.56	0	0.57 ± 0.14
Benzenoids (%)	1.93 ± 0.90	1.57 ± 0.86	16.58 ± 16.58	3.50 ± 3.40	0	8.23 ± 5.01	9.95 ± 9.95	2.76 ± 0.73
Terpenoids (%)	58.15 ± 18.84	17.03 ± 7.36	83.42 ± 16.58	84.63 ± 6.44	50.92 ± 20.41	39.02 ± 13.70	90.05 ± 9.95	96.67 ± 0.71
Monoterpenoids (%)	44.80 ± 17.03	9.38 ± 4.94	65.96 ± 20.01	52.03 ± 15.43	47.68 ± 19.73	35.94 ± 12.93	90.05 ± 9.95	5.64 ± 0.79
Sesquiterpenoids (%)	13.36 ± 4.52	7.64 ± 2.74	17.46 ± 14.04	32.60 ± 16.44	3.24 ± 2.07	3.08 ± 1.29	0	91.03 ± 1.34
Total N odour compounds detected	38	25	10	22	13	25	8	30
**Fatty acid derivatives**								
trans-4,8-dimethyl-1,3,7-nonatriene	4.64 ± 1.73	2.09 ± 0.72	0	7.53 ± 3.67	0	0	0	0.38 ± 0.17
cis-4,8-dimethyl-1,3,7-nonatriene	0.70 ± 0.36	0.12 ± 0.04	0	0.74 ± 0.74	0	0	0	0
2,4-dimethyl-1-heptanol	0	0	0	0	0.33 ± 0.24	0	0	0
Acetate (hexyl or heptyl)	0	0	0	0	0.67 ± 0.67	0	0	0
cis-3-hexenyl acetate	0.30 ± 0.30	53.67 ± 10.47	0	0	35.06 ± 21.48	8.59 ± 8.59	0	0
cis-3-hexenyl iso-butyrate	0	17.80 ± 2.67	0	1.78 ± 1.78	0	0	0	0
cis-3-hexenyl-2-methylbutanoate	0	5.04 ± 1.02	0	0	0	0	0	0

Unidentified unsaturated alcohols or alkenes	0	0	0	0	1.83 ± 1.47	0	0	0
Unidentified alcohol (C8)	0	2.26 ± 1.89	0	0.44 ± 0.44	0.04 ± 0.03	0	0	0
Unidentified aliphatic hydrocarbon	0	0	0	1.38 ± 0.88	1.64 ± 1.64	0	0	0.19 ± 0.07
Unidentified alkanes	34.26 ± 21.43	0	0	0	9.28 ± 6.47	44.16 ± 20.35	0	0
Unidentified ketone (C8)	0	0.41 ± 0.34	0	0	0.23 ± 0.23	0	0	0
**Benzenoids**								
2,6-Diisopropylnaphthalene	0	0	0	0	0	0	0	1.17 ± 0.48
Benzoic acid, methyl ester	0	0	0	3.38 ± 3.38	0	0	9.95 ± 9.95	0
Benzaldehyde	0.21 ± 0.09	0.03 ± 0.03	0	0.12 ± 0.08	0	0	0	0.09 ± 0.05
Benzene derivative (anisole?)	0.24 ± 0.14	1.54 ± 0.85	0	0	0	0	0	0
Bezeneacetaldehyde	1.32 ± 0.84	0	0	0	0	0	0	0
Cyclopentane (?)	0	0	16.58 ± 16.58	0	0	0	0	0
Pityol	0.17 ± 0.08	0	0	0	0	8.23 ± 5.01	0	1.50 ± 0.37
**Terpenoids**								
Cadinene(?)	0	0.39 ± 0.35	0	0	0	0	0	0
Cubebol (?)	0	0	0	1.63 ± 0.61	0	0	0	0
trans-β-farnesene	0	0	0	0	0	0	0	8.75 ± 1.48
1-(3-Ethylphenyl)ethan-1-one	0	0	0	0	0	0	0	0.96 ± 0.15

1-Phellandrene	0.45 ± 0.20	0.16 ± 0.11	0	0	0	1.89 ± 0.82	0	0
1,7-diepi-β-cedrene	0	0	0	0	0	1.05 ± 0.55	0	0
10s,11s-Himachala-3(12),4-diene	0	0	0	0	0	0	0	66.84 ± 1.6[illegible]
1H-cyclopropa[a]naphthalene	0.27 ± 0.10	0	2.74 ± 2.74	0	0	0	0	0
2,2-dimethyl-5-methoxyindan	0.28 ± 0.12	0	0	0	0	0.89 ± 0.83	0	0
3-Carene	6 .72 ± 2.89	5.63 ± 3.35	0	31.28 ± 12.84	46.99 ± 19.38	25.31 ± 10.07	35.45 ± 14.43	0.61 0.24
4-terpineol	0	0.45 ± 0.33	0	0.45 ± 0.37	0	0	15.06 ± 4.88	0.45 ± 0.20
α-selinene	0.47 ± 0.17	0	0	0	0	0.15 ± 0.13	0	0.63 ± 0.32
α-bergamotene	1.10 ± 0.47	0	7.01 ± 7.01	0	0	0.34 ± 0.25	0	0.31 ± 0.05
α-cubebene (?)	5.24 ± 3.60	0.40 ± 0.38	0	0	0	0	0	0
α-himachalene (?)	3.73 ± 3.56	0	0	0	0	0	0	0
α-longipinene	0	0	0	0	0	0	0	1.94 ± 0.13
α-terpinolene	1.08 ± 0.31	0	26.30 ± 24.60	0.22 ± 0.22	0	0	2.48 ± 0.72	0.22 ± 0.06
Aristolene	1.03 ± 0.52	6.11 ± 1.79	2.72 ± 2.72	28.86 ± 15.07	3.24 ± 2.07	0.63 ± 0.57	0	5.44 ± 0.60
β-myrcene	10.44 ± 5.37	0.15 ± 0.11	0	0	0	1.68 ± 0.68	0	2.71 ± 0.37
β-ocimene	5.20 ± 2.27	0.43 ± 0.27	0	12.06 ± 3.83	0.62 ± 0.62	1.53 ± 0.72	0	0
β-pinene	12.90 ± 4.91	1.71 ± 0.92	17.76 ± 10.37	1.51 ± 1.12	0	1.19 ± 0.52	0	0.14 ± 0.08
Camphene	0.28 ± 0.11	0	0	0	0	0	0	0.03 ± 0.02
Eucalyptol (?)	0	0	17.60 ± 15.25	3.40 ± 2.68	0.07 ± 0.07	1.02 ± 1.02	0	0.04 ± 0.03

Germacrene B	0	0	3.86 ± 3.86	0	0	0	0	0
Lilac aldehyde C	0	0	0	1.82 ± 1.82	0	0	29.05 ± 9.87	1.09 ± 0.60
Lilac aldehyde D	0	0.16 ± 0.14	0	0.32 ± 0.32	0	0	5.44 ± 2.20	0.03 ± 0.03
Linalool oxide	0	0	0	0.17 ± 0.17	0	0.53 ± 0.26	2.01 ± 0.67	0
Longifolene	0	0.15 ± 0.05	1.13 ± 1.13	0.94 ± 0.69	0	0	0	3.96 ± 1.19
Myrtanol (?)	0	0.67 ± 0.18	4.31 ± 3.71	0	0	0	0.55 ± 0.55	0.20 ± 0.11
Sabinene	7.08 ± 3.03	0.02 ± 0.02	0	0.22 ± 0.22	0	2.79 ± 1.19	0	0.05 ± 0.02
Unidentified monoterpenoids	0.65 ± 0.33	0	0	0.59 ± 0.28	0	0	0	0.08 ± 0.05
Unidentified sesquiterpenoids	0.53 ± 0.42	0.25 ± 0.25	0	1.17 ± 0.61	0	0.03 ± 0.03	0	2.19 ± 0.35
Zingiberene	0.70 ± 0.28	0.35 ± 0.32	0	0	0	0	0	0

Supplementary Table S2. Sterolic compositions from the pollen of the *Colletes succinctus* hosts, the *Melitta leporina* hosts and the non-host plants. The concentrations are expressed as percentage of total sterolic compounds. The three major sterols for each plant species are in bold.

Sterols	***Colletes succinctus* group**			***Melitta leporina* group**			**Outgroup**	
	Aster tripolium (n = 3)	*Calluna vulgaris* (n = 3)	*Hedera helix* (n = 3)	*Lythrum salicaria* (n = 3)	*Medicago sativa* (n = 3)	*Odontites luteus* (n = 3)	*Echium vulgare* (n = 3)	*Reseda lutea* (n = 3)
Cholesterol	2.10 ± 0.61	2.28 ± 0.52	4.40 ± 0.63	0.57 ± 0.11	1.73 ± 1.36	1.32 ± 0.23	1.50 ± 1.18	1.21 ± 0.02
Desmosterol	0.72 ± 0.57	0.05 ± 0.01	0.21± 0.07	0.06 ± 0.03	0.10 ± 0.06	N.D.	0.15 ± 0.24	0.12 ± 0.02
24-Methylenecholesterol/ campesterol	**19.80 ± 0.77**	2.16 ± 0.09	4.82 ± 0.12	**27.94 ± 0.44**	4.94 ± 4.94	**8.55 ± 5.09**	**65.62 ± 10.38**	7.31 ± 0.45
Stigmasterol	3.94 ± 2.53	**12.12 ± 0.96**	4.48 ± 0.01	0.86 ± 0.14	1.41 ± 1.64	4.62 ± 1.13	1.86 ± 0.52	4.66 ± 0.20
Unk.484	2.15 ± 0.26	1.47 ± 0.08	2.86 ± 0.38	1.91 ± 0.14	8.31 ± 0.62	5.55 ± 1.08	1.40 ± 0.31	3.34 ± 0.28
β-sitosterol	2.12 ± 0.37	**41.66 ± 1.42**	**24.70 ± 0.78**	**25.78 ± 0.38**	**10.22 ± 5.01**	**41.05 ± 2.07**	**12.95 ± 4.43**	**30.36 ± 1.19**
δ5-avenasterol	0.45 ± 0.21	**27.02 ± 0.58**	**12.68 ± 0.08**	**24.23 ± 2.51**	7.47 ± 4.60	**30.21 ± 1.75**	7.07 ± 3.17	**33.44 ± 2.36**
Cholestenone	1.67 ± 0.94	1.87 ± 0.33	1.90 ± 1.11	13.05 ± 1.72	2.23 ± 1.18	2 ± 0.45	5.80 ± 0.40	6.6 ± 1.53
δ7-stigmasterol	**40.34 ± 2.06**	2.44 ± 0.29	**32.12 ± 0.66**	4.77 ± 0.89	**53.81 ± 7.87**	4.93 ± 1.57	N.D.	**9.67 ± 0.15**
δ7-avenasterol	**26.70 ± 0.59**	8.94 ± 0.44	11.84 ± 0.41	0.83 ± 0.32	9.78 ± 5.31	1.76 ± 0.37	3.64 ± 2.70	3.29 ± 0.37

Supplementary Table S3. Amino acids from the host-pollen of the *Colletes succinctus* group, the *Melitta leporina* group and the outgroup. The concentrations are expressed as percentage of total amino acids.

Amino acids	***Colletes succinctus* group**			***Melitta leporina* group**			**Outgroup**	
	Aster tripolium (n = 3)	*Calluna vulgaris* (n = 3)	*Hedera helix* (n = 3)	*Lythrum salicaria* (n = 3)	*Medicago sativa* (n = 3)	*Odontites luteus* (n = 3)	*Echium vulgare* (n = 3)	*Reseda lutea* (n = 3)
Alanine	5.43 ± 0.04	5.07 ± 0.02	4.47 ± 0.03	5.33 ± 0.07	5.20 ± 0.05	4.65 ± 0.04	5.48 ± 0.04	4.90 ± 0.01
Arginine	5.17 ± 0.03	7.20 ± 0.04	6.00 ± 0.09	6.58 ± 0.10	5.96 ± 0.04	5.62 ± 0.10	6.39 ± 0.16	5.82 ± 0.11
Asparagine	9.77 ± 0.04	9.80 ± 0.04	9.77 ± 0.31	10.56 ± 0.07	9.85 ± 0.08	8.88 ± 0.34	10.44 ± 0.03	11.79 ± 0.11
Cysteine	1.32 ± 0.12	1.15 ± 0.05	0.99 ± 0.12	0.12 ± 0.08	0.95 ± 0.12	0.81 ± 0.12	0.27 ± 0.01	0.19 ± 0.02
Glutamate	11.06 ± 0.06	11.60 ± 0.13	9.86 ± 0.16	13.64 ± 0.12	11.82 ± 0.07	10.37 ± 10.18	11.52 ± 0.19	15.59 ± 0.17
Glycine	5.23 ± 0.04	4.36 ± 0.02	3.67 ± 0.03	4.28 ± 0.05	4.07 ± 0.01	3.82 ± 0.04	4.40 ± 0.05	4.13 ± 0.03
Histidine	5.38 ± 0.00	3.66 ± 0.01	2.91 ± 0.05	3.71 ± 0.05	3.41 ± 0.03	3.70 ± 0.02	3.63 ± 0.04	3.71 ± 0.05
Isoleucine	4.43 ± 0.01	4.78 ± 0.03	4.59 ± 0.05	4.82 ± 0.05	5.14 ± 0.04	4.87 ± 0.06	5.60 ± 0.05	4.71 ± 0.09
Leucine	7.09 ± 0.05	7.73 ± 0.02	6.72 ± 0.05	7.90 ± 0.08	7.81 ± 0.01	7.30 ± 0.10	7.75 ± 0.05	7.48 ± 0.10
Lysine	10.16 ± 0.05	7.92 ± 0.15	7.59 ± 0.09	8.03 ± 0.12	8.13 ± 0.10	7.35 ± 0.29	7.58 ± 0.10	7.50 ± 0.21
Méthionine	2.63 ± 0.14	2.95 ± 0.05	1.88 ± 0.71	0.50 ± 0.10	2.69 ± 0.15	2.46 ± 0.15	0.79 ± 0.02	0.37 ± 0.04
Phenylalanine	4.41 ± 0.02	5.07 ± 0.06	4.66 ± 0.08	5.18 ± 0.03	5.34 ± 0.08	4.81 ± 0.14	5.67 ± 0.02	5.01 ± 0.07
Proline	7.34 ± 0.08	7.98 ± 0.08	18.45 ± 0.16	8.77 ± 0.65	8.87 ± 0.03	16.38 ± 0.54	9.14 ± 0.65	8.41 ± 0.56
Serine	5.35 ± 0.02	5.59 ± 0.03	4.85 ± 0.06	5.40 ± 0.06	5.37 ± 0.03	4.87 ± 0.02	5.30 ± 0.04	5.36 ± 0.01
Threonine	5.46 ± 0.00	4.89 ± 0.03	4.58 ± 0.02	5.23 ± 0.07	5.04 ± 0.00	4.61 ± 0.02	5.83 ± 0.11	5.23 ± 0.07
Tyrosine	4.12± 0.05	4.29 ± 0.02	3.62 ± 0.06	4.07 ± 0.06	4.34 ± 0.08	4.07 ± 0.12	4.77 ± 0.02	4.01 ± 0.05
Valine	5.65 ± 0.09	6.07 ± 0.04	5.40 ± 0.11	5.89 ± 0.10	6.00 ± 0.06	5.44 ± 0.14	5.45 ± 0.07	5.81 ± 0.01

Supplementary Table S4. Location and coordinates of sampled host and non-host pollens

Bee group	Host-plant	Location	Coordinates
Colletes succinctus group	*Aster tripolium*	Netherlands, New-Namen, Saefthinge	51°21'32"N 04°13'06"E
	Calluna vulgaris	United Kingdom, Surrey, Milford	51°10'14"N 0°44'25"E
	Hedera helix	Belgium, Brussels, Koekelberg	50°51'36"N 04°19'58"E
Melitta leporina group	*Lythrum salicaria*	Belgium, Mons, University garden	50°27'55"N 03°57' 26"E
	Medicago sativa	Belgium, Mons, University garden	50°27'55"N 03°57' 26"E
	Odontites luteus	French, Gonfaron, Les Houerts des Maures	43°18´36"N 6°17' 46"E
Outgroup	*Echium vulgare*	Belgium, Mons, University garden	50°27'55"N 03°57' 26"E
	Reseda lutea	Belgium, Mons, University garden	50°27'55"N 03°57' 26"E
